# Commentary: Framing chronic absenteeism and emotionally based school absenteeism as public health problems

**DOI:** 10.3389/frcha.2026.1771056

**Published:** 2026-02-27

**Authors:** Arielle Symone Clarke, Kate Dimond Fitzgerald, Laura Mufson

**Affiliations:** 1 Division of Child and Adolescent Psychiatry, Department of Psychiatry, New York State Psychiatric Institute, New York, NY, United States; 2 Division of Child and Adolescent Psychiatry, Department of Psychiatry, Columbia University, New York, NY, United States

**Keywords:** chronic absenteeism, intervention, public health, racial-ethnic minority youth, school refusal

## 1 Introduction

The state of youth school absenteeism in the United States is troubling. Relative to a pre-pandemic absenteeism rate of 15% in 2019, U.S. Department of Education data indicates that 28% of students across elementary and secondary education were considered “chronically absent” in the 2022–2023 academic year (1, 2). Chronic absenteeism (CA)—absenteeism for at least 10% of school days—presents elevated risks for youth social disengagement, mental illness, legal system involvement, and long-term unemployment (3–7). As indicated in Kearney's recent Clinical Perspective on school attendance problems, CA has become a public health concern that will require an extensive, multisystem-level response that incorporates a full assessment of vulnerable populations and existing solutions to improve student outcomes (8).

Facing ever-widening disparities in CA post-COVID-19, racial-ethnic minority youth are among the populations most vulnerable to CA, navigating exacerbated structural inequities and intergenerational cycles of disadvantage (9, 10). While the adverse outcomes of general CA for at-risk youth are more well-documented (11–13), far less is known about the impact of emotionally-based absenteeism—also categorized and henceforth referred to as school refusal (SR)—within minoritized populations, a gap particularly pressing given pronounced post-pandemic increases in both global SR and minority youth depression, anxiety, and suicidality (14–16). Though grouped with CA in Kearney's article, effectively characterizing SR as a public health problem first necessitates an empirical understanding of how anxiety-based absenteeism manifests for under-resourced communities like Black, Indigenous, and People of Color (BIPOC). This commentary foregrounds why Kearney's public health approach to SR must account for BIPOC experiences so impactful solutions across tiers of public health intervention can be designed and implemented in diverse communities.

## 2 Understanding school refusal as a contributor to chronic absenteeism

SR is persistent school avoidance that is child-derived, anxiety-based, and typically accompanied by parental awareness and efforts to reduce child distress (e.g., parental accommodation). While at times conflated with SR both in research and policy, truancy is distinct, generally defined by education psychology scholars as unexcused absenteeism characterized by student nonengagement and delinquent behaviors, parental unawareness, and associations with externalizing disorders (e.g., conduct disorder) (17–20). Although SR prevalence is obscured by definitional ambiguity and limited research, estimates range from 1% to 2% of all school-aged youth and 5% to 16% in clinic-referred samples, with prevalence spikes in early childhood and early adolescence (21–23). Although 20% to 30% of these youth do not endorse psychiatric illness (22), SR is highly associated with internalizing mental health conditions (e.g., depression) (17), and may mediate well-established associations between CA and poor mental health outcomes associated with internalizing behaviors (e.g., youth suicidal ideation, reduced self-efficacy) (4, 24, 25).

## 3 To understand the trajectory of school refusal in racial- and ethnic-minority youth

Since studies have been largely conducted in predominantly White samples (26–30), SR remains poorly understood in BIPOC student populations. To date, no quantitative studies in the U.S. have explored the impact of race on SR progression, severity, and treatment (27, 29), hindering intervention tailoring for BIPOC children and adolescents. Nevertheless, a limited number of qualitative studies in Europe have examined the experiences of school-refusing ethnic minority and immigrant youth. For these youth, SR risk factors include parental pressures to succeed due to expectations of transgenerational upward mobility, the stress of navigating dual identities (i.e., identity within “home” and “host” cultural contexts), family-school communication/language barriers, and unfamiliarity with mental healthcare (31–33). Teachers unaccustomed to understanding youth from minoritized cultural backgrounds are less capable of managing their emotional distress and more likely to inaccurately profile their SR as oppositionality, perceiving the parents as unconcerned with their child's performance (31). Thus, the anxiety experienced by BIPOC youth may present a less urgent alarm to school staff regarding their attendance, resulting in delayed or otherwise absent referrals to appropriate services and an over-reliance on punitive school action (34, 35).

Navigating unique socio-economic barriers to school attendance, BIPOC youth experience psychosocial challenges that elevate potential for higher SR prevalence. Factors such as low socio-economic status (SES), inequitable access to general and specialized mental health services, disproportionate exposure to school disciplinary practices (i.e., suspension and/or expulsion), perceived racial-ethnic discrimination, and low-resourced school and home districts all likely contribute to SR for students of color (34–37). Given limited usage of mental health services and the underdiagnosis of internalizing disorders in these youth (38), it is likely that SR in BIPOC students goes undetected until it has become chronic and severe. Thus, the lack of inquiry into unique associations between psychosocial contributors and SR in BIPOC youth must be bridged to better design SR interventions.

## 4 Discussion

As demonstrated by Kearney, designating SR as a public health problem lends towards greater coordination in finding solutions (8). While emerging work suggests the utility of higher-tier multidisciplinary approaches that integrate mental health and education sectors in alignment with systems-based public health models (e.g., day programs such as In2School, LINK program) (8, 39–43), no clinical SR interventions have systematically considered racial-ethnic background and resource gaps. Given limited BIPOC engagement with care, a greater reliance on systems-based frameworks holds potential for improving intervention accessibility for youth otherwise left without proper SR identification and treatment. Expanding mental health supports in non-traditional settings wherein BIPOC youth may be more receptive (e.g., school-based mental health centers in low-SES districts, youth community centers, religious sites/churches, and telehealth/digital communication strategies) can facilitate early intervention while mitigating healthcare distrust (44–46).

Ecological public health frameworks analyze the influence of external (i.e., interpersonal, institutional, community, and structural) contexts on health outcomes (e.g., PRECEDE-PROCEDE model) (8, 39, 47). Considering SR under these models can spotlight the contribution of socio-economic obstacles to SR in BIPOC students, challenging prevailing clinical perspectives that frame SR as primarily individual (e.g., as solely motivated by student anxiety) by instead recontextualizing SR within cultural and structural factors (e.g., as motivated by anxiety reinforced by unsafe school districts or low school expectations of success) (48). For example, district-wide professional development trainings can be administered to improve teacher identification of SR risk factors and presentations in BIPOC youth, improving the ability to refer students to school-based clinics. Education and mental health leaders can work towards promoting climates less judgmental of BIPOC families and more focused on implementing a range of supports within schools (e.g., increase BIPOC school staff representation to improve cross-cultural home-school communication/collaboration), clinical care (e.g., incorporate caregiver and community perceptions towards education into therapeutic work), and policy domains (e.g., increase funding for mobile crisis service programs and streamlined transportation capabilities in low-resourced home/school districts).

Implementing culturally-tailored evidence-based programming within a comprehensive public health approach requires critical empirical inquiry by researchers into the experiences of BIPOC youth. Clinical and implementation researchers must study the utility and effectiveness of interventions for BIPOC SR, their implementation in diverse settings, and their eventual incorporation into state policy. Accordingly, targeted recruitment of minoritized children and adolescents is needed to promote a greater degree of culturally-responsive and racial trauma-informed treatment considerations (e.g., culture-specific family dynamics, discriminatory school environments, mental health stigma) among mental health providers, education professionals, and policy-makers alike, thereby improving the quality of care for these vulnerable youth.
